# Intrasinus Thrombolysis for Cerebral Venous Sinus Thrombosis: Single-Center Experience

**DOI:** 10.3389/fneur.2019.01185

**Published:** 2019-11-13

**Authors:** Xinbin Guo, Jiachen Sun, Xiaoke Lu, Sheng Guan

**Affiliations:** Department of Interventional Radiology, The First Affiliated Hospital of Zhengzhou University, Zhengzhou, China

**Keywords:** cerebral venous sinus thrombosis, intrasinus thrombolysis, safety, validity, anticoagulant

## Abstract

**Objective:** The purpose of this research was to study the safety and efficacy of intrasinus thrombolysis in patients with cerebral venous sinus thrombosis unresponsive to conventional heparin therapy.

**Methods:** A total of 156 CVST patients were treated using interventional thrombolysis in our center from January 2010 to June 2018. Clinical data, including duration of symptoms, indications and outcome of IST were retrieved, and outcomes were analyzed. DSA or MRV was used to assess the recanalization after thrombolysis. mRS was used to evaluate the outcome at admission, discharge, and follow-up.

**Results:** 91.38% of patients obtained functional independence (mRS 0–2). The mRS score was 0–2 in 120 patients (76.92%, 120/156) at the time of discharge. Seven patients succumbed during hospitalization. MRV examination was performed in 149 patients, and the results showed that the venous sinus of 112 patients (75.17%) was completely recanalized, and it was partially recanalized in 28 patients (18.79%) and nine patients (6.04%) had no recanalization at the time of discharge. In total, 116 patients were followed up at least for 6 months, 89 patients (76.72%) were completely recanalized, 21 patients (18.1%) were partially recanalized, and six patients (5.17%) were not recanalized.

**Conclusion:** IST may be more effective than systemic heparin anticoagulation in moribund and unresponsive patients despite the risk of hemorrhage. Large randomized controlled trials are required to further evaluate this issue.

## Introduction

Cerebral Venous Sinus Thrombosis (CVST) is an uncommon disease with an annual incidence of 0.5–1%, but could cause potentially life-threatening ischemic stroke, especially among young women (1, 2). Typical clinical symptoms of CVST include headaches, blurred vision, limb paralysis, consciousness disorders, and coma. Due to the various clinical presentations, the misdiagnosis rate of CVST is relatively high and the mortality rate of severe CVST is 10–30% (1, 3, 4).

Anticoagulant therapy is the first line treatment for CVST (4, 5). Although most patients respond very well to treatment with heparin, some patients might undergo an adverse course with subsequent worse outcomes. The subgroup of patients with poor outcome are those with coma, intracerebral hemorrhage (ICH), rapidly progressing clinical deficits, and involvement of the deep venous system (6, 7). Approximately 30% of patients with one or more of these risk factors had poor outcome despite the treatment with heparin (8). Endovascular intrasinus lysis might yield better outcome in these patients (9, 10). Identifying the patients with severe clinical grade at early stage and administration of endovascular lysis in this subgroup result in favorable outcome.

We have previously presented our series of 37 patients in 2012, who received LIST in our institution and showed that local thrombolytic therapy was beneficial for the recanalization and consequent reversal of neurological deficits in CVST (11). To further identify the efficacy of local interventional thrombolysis on CVST, our current retrospective study analyzed the outcome of 156 patients, focusing on the treatment safety and efficacy in our center. To date, our study is the largest series of IST in CVST reported from China.

## Subjects and Methods

### Patients

This study was approved by the ethics committee of Zhengzhou University. All work described has been carried out in accordance with The Code of Ethics of the World Medical Association. Informed written consent was obtained from all individual participants included in the study.

From January 2010 to June 2018, the clinical and angiographic data of all patients for CVST treated with IST were reviewed at our institution −156 patients diagnosed as severe CVST received local thrombolysis in the Department of Neuro-interventional Radiology. CVST was confirmed by magnetic resonance imaging (MRI)/magnetic resonance venography (MRV) and digital subtract angiography (DSA). Anticoagulation with conventional heparin was the initial treatment in all the patients. The anticoagulation treatment consisted of heparin 1 Ug/kg intravenous and bolus dosing given twice a day. Partial thromboplastin time (PTT) monitoring was performed to adjust the dose of heparin. Patients who were offered intrasinus thrombolysis were those who satisfied the following criteria: (1) symptoms have not been significantly improved or neurological functions are rapidly deteriorated after anticoagulation treatment before interventional thrombolysis; (2) Glasgow Coma Score (GCS) ≤ 10 points at admission or during treatment; (3) straight sinus thrombosis or large area cerebral infarction with poor prognosis; (4) an assumed poor prognosis because of an altered mental status (cognitive disturbances including abnormal alertness and orientation, coma), straight sinus thrombosis, or large space occupying stroke; and (5) patient consent was obtained before the procedure. The exclusion criteria were as follows: (1) anticoagulant therapy was effective and the symptoms significantly relieved; (2) significant cardiac, liver or renal dysfunction; (3) patients with a large hematoma that caused herniation; (4) CVT secondary to malignancy such as leukemia; and (5) patients refused interventional thrombolysis.

### Procedures

The “Seldinger” technique was used for right femoral artery puncture, and a 5F diagnostic catheter (Terumo Corporation, Japan) was used to perform a cerebral arteriogram to evaluate venous outflow pathways after systemic heparinization. A 6F guiding catheter (Terumo Corporation, Japan) was placed into the jugular bulb or sigmoid sinus through the right femoral vein, and the microcatheter was introduced into the distal end of the thrombosed sinus via the internal jugular vein under the guidance of a guidewire. Continuous urokinase (42,000 U/h, total 1,000,000 U per day) was administered into the cerebral venous sinus by microcatheter, and heparin was continuously administered through the guiding catheter to maintain systemic heparinization. The microcatheter was withdrawn 2–3 cm after 2–3 days. On the 5th day of thrombolysis, DSA or MRV was performed to observe the CVST recanalization. If the CVST was not recanalized, the position of the micro-catheter would be adjusted and urokinase was to be continued. Infusion was continued until significant clinical improvement or partial recanalization of the sinus with good outflow were obtained. All patients in the thrombolysis group were subsequently started on oral anticoagulation for 6 months (warfarin therapy), and International Normalized Ratio (INR) was maintained between 2.0 and 3.0.

### Clinical Events

Any clinical events during the thrombolysis course were noted. Neurologic assessment and a related image exam were performed before the treatment, at discharge and at follow-up.

### Clinical Assessment and Follow-Up

The recanalization of CVST patients was evaluated by MRV at discharge and follow up for 6 months. The criteria for recanalization were as follows: (1) complete recanalization was that all the occluded sinus development was recanalized under DSA or MRV; (2) partial recanalization was defined as the complete recanalization of one sinus but persistent occlusion of other sinuses, or one or more sinuses were partly recanalized; and (3) no recanalization was defined as that all the occluded sinuses failed to reach recanalization. Clinical outcome was measured using modified Rankins score with scale 0, normal; 1, no significant disability; 2, slight disability (look after own affairs without assistance); 3, moderate disability (need help, able to walk when assisted); 4, moderately severe disability (unable to walk unassisted); 5, severe disability (unable to ambulate, altered mentation); 6, death. Efficacy can be divided into good outcomes (mRS 0–2), partial improvement (mRS 3–4 points) and poor outcomes (mRS 5–6 points).

### Data Analysis

Continuous variables are expressed as median (range) and qualitative variables as count (percentage).

## Results

### Clinical Manifestations and Sinus Involvement

**Figure 1:**
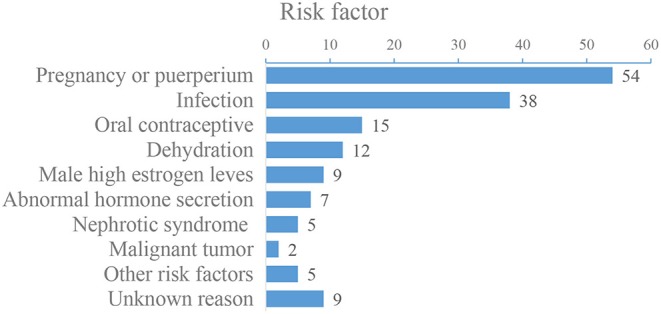
Risk factors of 156 patients with severe CVST.

**Figure 2:**
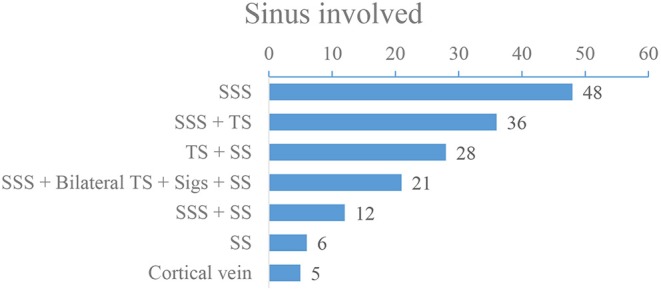
The involvement of CVST in 156 patients with severe CVST.

Of the 156 patients, 37 patients were male and 119 patients were female, the mean age of the patients was (32 ± 5) years old (ranging from 14 to 68 years old). Headache was the most common symptom, and the main risk factors were pregnancy or puerperium (Figure 1). Baseline demographic characteristics and clinical manifestations are shown in Tables 1, 2, and risk factors are shown in Figure 1. DSA showed that 59 (37.82%) patients had a single venous sinus involved, and 97 (62.18%) patients had two or more sinuses involved, superior sagittal sinus (SSS) and transverse sinus (TS) are most commonly affected (Figure 2).

**Table 1:** Baseline demographic characteristics.

**Baseline demographic characteristics**	**Numbers**
Sex (*n*, %)	
Male	37 (23.7%)
Female	119 (76.3%)
Age (mean ± SD, range, years)	32 ± 5 (14–68)
Hypertension	108 (69.23%)
Diabetes mellitus	31 (19.9%)
Gestation (to female)	47 (39.5)
Smokers	28 (17.9%)
Drinkers	39 (25%)

**Table 2:** Clinical manifestations of 156 patients with severe CVST.

**Clinical manifestation**	**Numbers**
Headache with nausea and vomiting	135 (86.54%)
Hemiparalysis	83 (53.21%)
Epilepsy	14 (15.38%)
Disorder of consciousness	17 (10.90%)
Sensory disorder	16 (10.26%)
Vision disorder	15 (9.62%)
Aphasia	14 (8.66%)
Coma	9 (5.51%)

### Sinus Recanalization

All patients underwent intrasinus thrombolysis. There were no major procedure-related complications in any of the patients. A total of 132 patients (84.61%) received SSS catheter thrombolysis, and 24 cases (15.39%) received SSS and straight sinus (SS) double-microcatheter thrombolysis; the duration of thrombolysis was about 5–7 days. All patients had a history of heparintherapy. Seven patients died during hospitalization. MRV examination was performed in 149 patients after thrombolysis, and the results showed that the venous sinus of 112 patients (75.17%) was completely recanalized, partial recanalization was seen in 28 patients (18.79%) while nine patients (6.04%) had no recanalization at discharge.

A total of 116 patients (74.36% of all subjects) were followed up at 6 months. MRV showed that 89 patients (76.72%) had completely recanalized, 21 patients (18.1%) had partially recanalized, and six patients (5.17%) showed no recanalization during follow up at 6 months (Table 3). Two out of the six completely occluded patients had symptoms of cranial hypertension and continued to take oral warfarin with International Normalized Ratio (INR) maintained between 2.0 and 3.0.

**Table 3:** Sinus recanalization rate after thrombolysis, discharge and 6 months follow-up.

	**Completely recanalization (%)**	**Partial recanalization (%)**	**No recanalization (%)**
After thrombolysis (*n* = 156)	58.97	33.33	7.69
Discharged (*n* = 149)	75.17	18.79	6.04
Follow-up (*n* = 116)	76.72	18.1	5.17

### mRS Outcome

The patient's clinical prognosis was assessed at admission, discharge and follow-up at 6 months. Two patients developed intracerebral hemorrhage during intrasinus thrombolysis. Local thrombolysis was stopped, but they had continued systemic anticoagulation. Both of those patients had a good clinical outcome. The mRS score was 0–2 in 120 patients (76.9%) at discharge and 3–4 in 25 patients (16.03%). A total of 11 patients (7.05%) had mRS scores of 5–6, among which three patients had large infarction and intracranial edema, and were eventually transferred to the surgical department for further treatment. Three patients had severe intracranial hemorrhage at admission and the situation progressed rapidly; eventually the patients passed away. Two patients were found to have malignancy and they subsequently passed away 3 weeks later. Two patients were diagnosed with lupus encephalopathy and also subsequently died during hospitalization. The main risk factor profiles of the 11 patients with poor outcome are shown in Table 4.

**Table 4:** The main risk factors for 11 poor outcome patients.

**Risk factor**	**No. of patients (%)**
Hemorrhage	3 (27.3%)
Hemorrhagic infarct	3 (27.3%)
Cancer cells in GCF	2 (18.2%)
lupus encephalopathy	2 (18.2%)
Deep venous system thrombosis	1 (9.1%)

**Figure 3:**
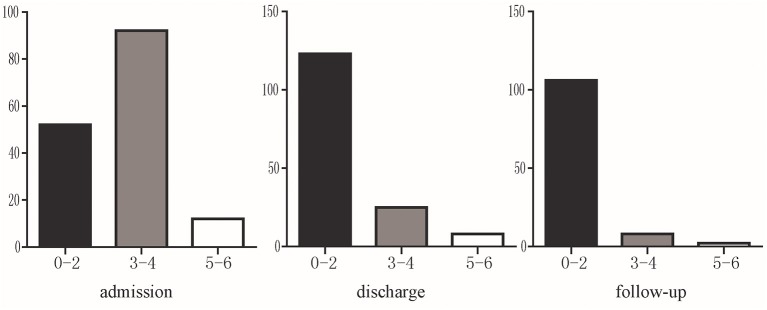
The mRS of severe CVST at admission, discharge and 6 months follow-up. Black indicates significant improvement (mRS 0–2); gray indicates residual neurological symptoms (mRS 3–4); white indicates poor prognosis (mRS 5–6).

A total of 116 patients were followed up at 6 months. Among them, 91.38% (106/116) patients had a mRS score of 0–2, 6.90% (8/116) had a mRS score of 3–4, and 1.72% (2/116) were dead during follow-up (mRS 6 points). mRS distribution is shown in Figure 3.

### Illustrative Cases

#### Case 1

**Figure 4:**
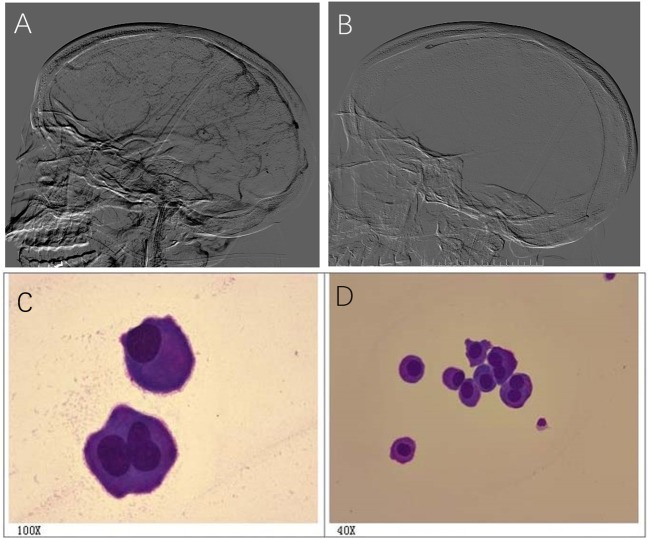
A 36-year-old female had a progressive headache and cerebrospinal fluid cytology revealed a large number of abnormal cells. **(A)** DSA revealed thrombosis in the SSS, SS,and TS; **(B)** Image of intrasinus thrombolysis; **(C,D)** Cerebrospinal fluid cytology revealed abnormal cells, and considered malignant tumors **(C,D)**.

A 36-year-old female had a progressive headache. The patient was diagnosed with CVST by MRV and treated with anticoagulant therapy with no response to systemic heparinization. DSA revealed thrombosis in the SSS, SS, and TS (Figure 4A). Lumbar puncture showed intracranial pressure >600 mmH_2_O. On the second day after admission, intrasinus thrombolysis was performed (Figure 4B). During thrombolysis, the patient's symptoms gradually got worse and lost vision gradually. Cerebrospinal fluid cytology revealed a large number of abnormal cells, and considered malignant tumors (Figures 4C,D). On the 8th day after thrombolysis, the patient had sudden consciousness disturbance and died in emergency.

#### Case 2

**Figure 5:**
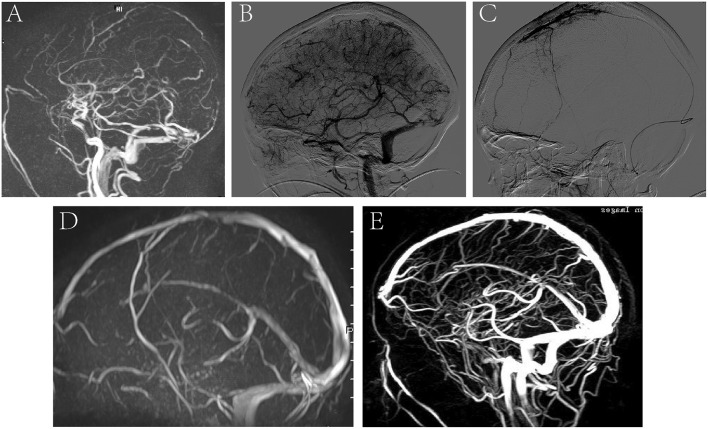
A representative image of a severe CVST before and after thrombolysis, and at 6 months follow-up. **(A)** MRV image showed CVST; **(B)** DSA showed no recanalization of the SSS and SS before treatment; **(C)** microcatheter was placed in the SSS. **(D)** MRV showed the SSS and SS were recanalized at discharge after thrombolysis. **(E)** MRV showed venous sinus were unobstructed and there was no significant recurrence at 6-month follow-up.

A 35-year-old pregnant woman presented with headache and nausea for 4 days, physical convulsions, and low right limb muscle strength (I–II). MRV showed CVST and the intracranial pressure was 300 mmH_2_O (Figure 5A). DSA showed no recanalization of the SSS and SS before treatment (Figure 5B), and microcatheter was placed in the SSS (Figure 5C). On the 5th day of thrombolysis, the symptoms of the patients completely resolved. MRV showed the SSS and SS were recanalized at discharge (Figure 5D). MRV showed venous sinus were unobstructed and there was no significant recurrence at 6-month follow-up (Figure 5E).

## Discussion

CVST is a rare type of cerebral stroke which may be presented as a variety of clinical symptoms and can be easily misdiagnosed due to the non-specific diagnostic methods. The mortality of CVST is about 10–30% (4). Studies have shown that early diagnosis and effective treatment are critical to the prognosis of CVST, which could reduce mortality to 5–15% (1, 12).

Anticoagulant therapy was the basic therapeutic method of venous sinus thrombosis treatment. However, anticoagulant therapy was not always effective for all CVST (13–15). For the treatment of CVST, the occluded venous sinus should be recanalized in a timely fashion to prevent the progression of the disease. The hypothesis of local intrasinus thrombolysis relies on increasing the therapeutic concentration to reach the peak at the location of CVST, so that the occluded venous sinus could be recanalized rapidly, and the stagnation of venous blood was alleviated, to avoid further neurological deterioration (16). One study of 169 patients with CVST indicated that 48% of patients had complete recanalization after interventional thrombolysis (17). In a systematic review of interventional therapy for CVST, the occluded venous sinus of 69% of patients was completely recanalized after local thrombolysis, and the occluded venous sinus of 26.3% patients was partially recanalized. A total of 76% of patients had one to mild clinical symptoms (mRS 0–2), 9.6% patients had moderate clinical symptoms (mRS 3–4), 1.2% patients had severe clinical symptoms (mRS 5) and 13.2% patients died at long-term follow-up (mRS 6) (4–18). The data of our institution showed that 76.72% of patients had complete recanalization of venous sinus, and 91.38% of patients had venous sinus interventional thrombolysis with mRS score of 0–2 at follow-up. Our results indicated that the clinical prognosis was closely related to the recanalization of CVST and interventional thrombolysis was effective in the treatment of CVST.

Currently, the most common complication of thrombolytic therapy for CVST is intracranial hemorrhage (2, 7), which is caused by elevated intra-sinus pressure and associated venous vascular wall rupture (19). Local thrombolysis could recanalize CVST rapidly and reduce the intracranial pressure as well as the risk of intracranial hemorrhage. Intracranial hemorrhage was not a contraindication to interventional thrombolysis. Recently, a study consisting of 10 cases of hemorrhagic CVST underwent with interventional thrombolysis showed that only one patient died 48 h after thrombolysis (20). In our study, the incidence of hemorrhagic complications was 3.85%. A total of 17 patients had intracranial hemorrhagic on the time of admission, and three of them had progressive hemorrhage during treatment and eventually died. In addition, six patients had no intracranial hemorrhage at admission, and intracranial hemorrhage happened during thrombolysis. With the development of imaging technology and interventional technology, most patients with CVST could be diagnosed timely and received standardized, evidence-based and effective treatment. During the thrombolytic process, the dose of urokinase was adjusted correspondingly to further improve the safety of thrombolytic therapy according to the patient's coagulation function, clinical manifestations and so on.

In conclusion, our study highlights the safety and benefit of direct thrombolytic infusions, particularly in patients unresponsive to anticoagulation. Further investigation should include recruiting randomized controlled trials and making more individualized treatment plans according to each patient's situation.

## Data Availability Statement

The datasets generated for this study are available on request to the corresponding author.

## Ethics Statement

The studies involving human participants were reviewed and approved by the ethics committee of Zhengzhou University. The patients/participants provided their written informed consent to participate in this study.

## Author Contributions

XG, XL, and JS performed neuroimaging and intervention in this clinical trial. SG and XG wrote the main manuscript text.

### Conflict of Interest

The authors declare that the research was conducted in the absence of any commercial or financial relationships that could be construed as a potential conflict of interest.
